# Role of Intraoperative Esophagogastroenteroscopy in Minimizing Gastrojejunostomy-Related Morbidity: Experience with 2,311 Laparoscopic Gastric Bypasses with Linear Stapler Anastomosis

**DOI:** 10.1007/s11695-012-0757-2

**Published:** 2012-09-03

**Authors:** Ashraf Haddad, Nicholas Tapazoglou, Kuldeep Singh, Andrew Averbach

**Affiliations:** Saint Agnes Hospital, Baltimore, MD USA

**Keywords:** Intraoperative esophagogastroenteroscopy, LRYGB, Gastrojejunostomy leak rate

## Abstract

**Background:**

Anastomotic leaks and strictures of the gastrojejunostomy are a cause of major morbidity following laparoscopic Roux-en-Y gastric bypass (LRYGB). Reported rates of leaks vary between 0 and 5.2 %. This has led bariatric surgeons to use a variety of intraoperative methods to detect incompetent suture lines. The aim of the study was to evaluate the role of intraoperative endoscopy in reducing the rate of postoperative anastomotic complications. The setting of this study is in a community teaching hospital.

**Methods:**

Medical records of 2,311 patients who underwent a LRYGB from 2002 to 2011 were retrospectively reviewed utilizing the hospitals’ bariatric surgery database. Demographics, weight, body mass index, intraoperative endoscopy results, and postoperative outcomes within 90 days after surgery were analyzed.

**Results:**

Endoscopy was attempted in 2,311 patients and completed in 2,308 (99.9 %). Intraoperative leak was detected in 80 (3.5 %) patients; suture line was reinforced in 46 patients (2 %), while in the other 34 patients the leak was transient at only high insufflation pressure. Postoperative clinical leaks were detected in four cases (0.2 %) two of which had initial leaks intraoperatively. In two cases, the anastomosis was too tight and required reconstruction. Twenty-five patients (1.1 %) developed early postoperative strictures requiring endoscopic dilatation within 90 days. Three patients (0.1 %) had iatrogenic injury at the time of intraoperative endoscopy, all three healed without delayed morbidity.

**Conclusions:**

The routine use of intraoperative endoscopy in LRYGB with the linear stapler anastomosis technique is associated with a complication/failure rate of 0.3 % and low gastrojejunostomy-related morbidity after LRYGB within 90 days (leak rate of 0.2 % and stricture rate of 1.1 %).

## Introduction

Laparoscopic roux-en-y gastric bypass (LRYGB) is a technically challenging procedure, which is performed frequently in the USA and is becoming more popular in other countries [1]. There are several techniques of gastrojejunal anastomosis (GJA) construction with linear stapler and partially hand-sewn anastomosis being one of them. Anastomotic leak is one of the most serious potentially preventable complications with reported rates of 1–5 % [2]. Current guidelines of the American Society of Metabolic and Bariatric Surgery (ASMBS) (2009) state that “the vast majority of gastrointestinal leaks occur in the absence of technical error” and “no high-quality clinical evidence exists that intraoperative technique is able to eliminate or substantially decrease the incidence of leaks as a complication of gastric bypass”. Nevertheless, GJA leaks remain a cause of major morbidity and every effort to decrease it is justified [3, 4]. In this retrospective study, an attempt was made to evaluate the role of the routine use of endoscopy in reducing GJA-related morbidity.

## Patients and Methods

A retrospective review involved analysis of the medical records of 2,311 consecutive patients with LRYGB performed in 2001 to 2011. Patient’s database was reviewed for sex, weight, body mass index (BMI), postoperative complications, and length of hospital stay. The database included follow-up data from the surgeons’ offices on 90-days morbidity, mortality, and therapeutic interventions.

Of the 2,311 patients 1,849 were females (80 %) with an average age for both sexes of 44 ± 10.3 years. The average BMI was 49.8 ± 8.3. Demographic data are summarized in Table 1.

**Table 1:** Demographic data of patients

Gender	Number/range	% of all patients/average ± SD (standard deviation)
Females	1,849	80.00 %
Males	462	20.00 %
Age (years)	18–69.75	44 ± 10.3
BMI (body mass index)	32.1–114.5	49.8 ± 8.3

## Operative Technique

All procedures were performed in integrated minimally invasive operating rooms with laparoscopic and endoscopic capabilities by a bariatric surgeon (KDS or AMA) with the assistance of a senior surgical resident and one surgical assistant. Standardized procedure technique involved construction of the proximal gastric pouch, followed by construction of the Roux limb in a retrocolic, retrogastric position and a gastrojejunostomy between the posterior wall of the proximal gastric pouch and the antimesenteric aspect of the Roux limb. Prior to GJA construction, the Roux limb was secured to the proximal gastric pouch with the blind end facing left and checked for axial rotation from within the omental bursa and from the infracolic aspect. The gastrojejunostomy was fashioned in two layers with the outer posterior layer created first with running 2.0 Surgidac Endo Stitch and the inner layer partially stapled with EndoGIA—45 and 2-cm length of the staple line. The inner layer was completed with running 2.0 Surgidac Endo Stitch with purse-stringing of the anastomosis to a diameter of about 15 mm. At completion, it assumed an oval to round shape. Following that, the anterior sero-muscular layer was finished with running 2.0 Surgidac Endo Stitch.

Upon completion of the anastomosis, the Roux limb was clamped with a bowel clamp about 5 cm distally. The table was leveled and the left subdiaphragmatic space was filled with sterile normal saline to cover the proximal pouch and anastomosis. The area was irrigated and aspirated repeatedly until the irrigant became clear from blood and debris. Intraoperative endoscopy was performed by the attending surgeon or senior resident. The gastroscope was advanced with the controls in the unlocked position posterior to endotracheal tube at 5:30 or 6:30 o’clock position and was introduced under digital control without force across the superior esophageal sphincter. Occasionally a “jaw thrust” maneuver provided by the anesthesiologist was required to assist in advancement of the instrument. In case of persistent difficulty with insertion of the endoscope, superior laryngeal structures were visualized with insufflation for appropriate guidance of the instrument. Subsequent advancement of the instrument was done under direct visualization. The proximal pouch was examined and then the gastroscope was negotiated across the anastomosis into the Roux limb. The gastroscope was pulled back into the proximal pouch and the anastomosis re-inspected with continuous insufflation. Following that, the jejunum was accessed again and then all compartments were desufflated while withdrawing the gastroscope making sure no substantial amount of air is left. In case of persistent air leak, the gastroscope was left in position and the procedure was repeated after repair or reinforcement of gastrojejunostomy suture line.

After completion of endoscopy, the Roux limb was secured in the mesocolic window and Peterson’s defect was closed with two running 2.0 Surgidac Endo Stitches. A Jackson Pratt drain was routinely placed and positioned posterior to the anastomosis.

An upper gastrointestinal (GI) gastrograffin imaging was performed on the first postoperative day. Patients were started on clear liquid diet and advanced to a pureed diet on the second day. The drain was removed and the patients were usually discharged on the second postoperative day unless there were clinical indications for further observation. Sampling of drain fluid for amylase on the second postoperative day was utilized liberally if there were any concerns regarding the gastrojejunostomy integrity.

## Results

**Fig. 1:**
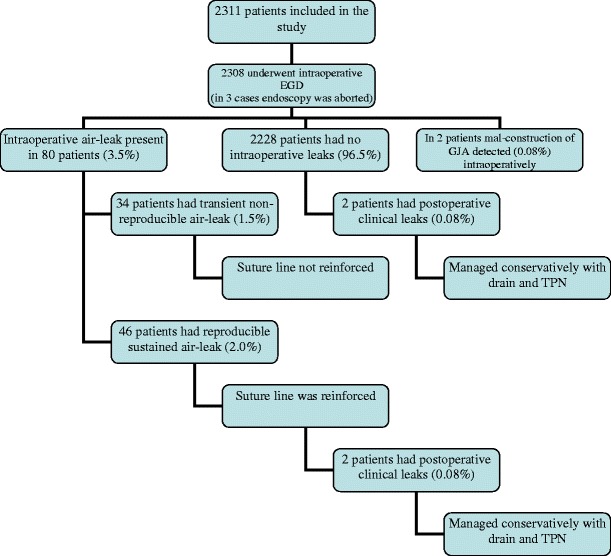
Distribution of study patients by results of intraoperative esophagogastroenteroscopy and postoperative anastomotic leak rates. *Abbreviations*: *EGD* esophagogastroenteroscopy, *GJA* gastrojejunal anastomosis

Intraoperative esophagogastroenteroscopy (EGD) could not be completed in three (0.1 %) patients due to technical inability to pass the instrument into the esophagus because of extreme redundancy of soft tissues or tight superior esophageal sphincter (Fig. 1). Three (0.1 %) patients sustained iatrogenic injury at the time of intraoperative endoscopy. One patient had sustained a proximal gastric pouch tear due to the locked position of the controls of the gastroscope. Injury was recognized and repaired intraoperatively. The other two patients had pharyngeal tears; one was detected intraoperatively and the other one on the postoperative UGI series. The pharyngeal tears were managed conservatively with TPN and NPO for 7 days without delayed morbidity. Total failure and morbidity rate of intraoperative endoscopy was encountered in six of 2,311 patients (0.26 %).

Intraoperative air leaks were detected in 80 (3.5 %) patients. In such instances, the proximal gastric pouch and the anastomosed portion of the jejunum were desufflated and reinsufflated repeatedly while accessing the jejunum to determine if there is a sustained high-volume air leak. Transient leaks with high insufflation pressure occurred with initial insufflation and were associated with small air bubbles. If there was no sustained air leak, no further action was taken. In 46 patients (2.0 %), the air leak was persistent. With partial aspiration of the irrigant, attempts were made to determine its location and then the anterior or posterior lip of the gastrojejunostomy suture line was reinforced with 2.0 Surgidac Endo Stitch. Following that the anastomosis was tested again.

Postoperatively, clinical leaks were detected in four cases (0.2 %). Two of which had an intraoperative air leak. In one out of four patients, the leak developed after the reconstruction of the GJA which was required on postoperative day 2 due to a diagnosed axial rotation of the Roux limb within the mesocolic window causing Roux limb obstruction. The presentation of patients with GJA leaks included fever, mild tachycardia, slight leukocytosis, nausea and/or increased drain output. In all cases, an UGI series with gastrograffin was repeated and a drain fluid amylase level was studied. In the case of a confirmed leak, drain fluid amylase levels were always in a range of several thousand units. All four leaks were detected by postoperative day 3. They were managed conservatively with the previously placed drains and total parenteral nutrition and healed within 4–6 weeks. None of the patients with clinical leak required surgical intervention. All patients were females. There was no postoperative GJA-related mortality.

In two cases, the gastroscope could not be passed into jejunum due to overtightening of the inner layer of the suture line (one patient) and due to a mucosal flap secondary to introducing the stapler in the gastrostomy between the layers of the gastric wall (one patient). In both cases, the gastrojejunostomy was immediately revised without postoperative complications.

In the postoperative period, 25 (1.1 %) GJA strictures were detected within 90 days. Patients mostly presented at weeks 3 to 6 after the initial procedure once regular diet was introduced. All patients underwent a single endoscopic pneumatic balloon dilatation with 18–20-mm balloon without further delayed intervention. There were no delayed strictures at 1-year follow-up. There was no correlation in occurrence of GJA related morbidity to age, gender, BMI, or co-morbidities. Average length of hospital stay was 2.0 days (range, 1–87 days).

Total GJA-related postoperative morbidity has occurred in 31 of 2,311 patients (1.3 %) and included 2 patients with faulty GJA construction detected and repaired intraoperatively, 4 postoperative leaks, and 25 early strictures. Assuming intraoperative endoscopy was not utilized, there would have been 50 cases of anastomosis related morbidity excluding strictures (46 cases of large volume air leak intraoperatively, 2 leaks in the endoscopy negative group, and 2 cases of GJA misconstruction (0.2 %)). With 25 cases of strictures, that would have brought postoperative GJA related morbidity to 75 case or 3.2 % of all 2,311 patients.

## Discussion

Postoperative GJA leaks are one of the most serious complications after LRYGB and every possible step should be taken to avoid them. In the literature, reported leak rates range from 0 % to as high as 5.2 % [2–6]. While in the ASMBS position statement it is advised that “the vast majority of GI leaks likely occur in the absence of technical error …” we believe that the frequency of this complication is potentially modifiable.

Fernandez et al. (2004) analyzed the risk factors of postoperative morbidity in more than 3,200 patients that underwent gastric bypass and identified weight, hypertension, and the type of bypass (revision > open > laparoscopic), and GJA leak as independent risk factors associated with post operative mortality. They reported age and male gender as independent risk factors for developing leaks. Also, patients who developed a leak were noted to have a higher incidence of diabetes and sleep apnea. Interestingly, in the 554 LRYGB subgroup, the only significant risk factor for developing leaks was diabetes (*P* value 0.0284) [7].

Experience and the learning curve of the surgeon and their relation to the development of leaks has been the subject of several studies. While Gonzalez et al. [8] reported no relation between the number of procedures and leaks, Schauer and colleagues [9] suggested a decrease in operative complications with increasing experience.

Symptoms of postoperative leaks are non-specific and include, fever, tachycardia, hiccups, nausea, and abdominal pain. It is well-known that such clinical symptoms are difficult to interpret early in the course of this complication and/or are possibly absent in the morbidly obese patient. It was suggested based on experience with pharyngeal and pancreatic surgery that drain fluid amylase level after esophagoenterostomy (predominantly salivary amylase) can be utilized to diagnose anastomotic leaks [10]. Maher and colleagues demonstrated that drain amylase level is a low-cost adjunct with high sensitivity and specificity that helps in detecting anastomotic leaks early on before a septic picture develops [11]. In our experience, assessment of drain amylase was helpful in confirming the anastomotic leak early while the drain output still remained serous and might just have increased in volume. Combination of UGI series and drain fluid amylase testing allowed the diagnosis of all four leaks by postoperative day 3 as well as the institution of conservative therapy early while the patient is in a stable condition. No re-exploration was undertaken and management of this serious complication was simplified.

Upper GI water-soluble contrast study is usually obtained on postoperative day 1 in laparoscopic cases, a fact that might have led to quicker detection of leaks in laparoscopic vs. open cases (median 1 vs 3 days) [4]. Madan et al. (2007) reported positive and negative predictive values for UGI of 67 and 99 %, respectively, in 245 patients who underwent LRYGB. This negative predictive value was only superseded by elevated WBC count > 10.5 that had a negative predictive value of 100 % for leaks [12]. It is known that UGI might be falsely negative and miss small subclinical leaks and the presence of leukocytosis is also variable. Some authors questioned the usefulness of routine drain placement and upper GI series after gastric bypass procedures, but had to utilize diagnostic laparoscopy and endoscopy to address suspected anastomotic problems [13, 14]. In general, routine use of intraoperative endoscopic assessment of GJA coupled with upper GI series and drain placement is probably a less involved management strategy and provides a simpler approach for both the patient and treating surgeon. Again, none of our patients with GJA leaks required additional surgical intervention.

In an attempt to reduce the incidence of postoperative leaks, several methods were suggested to evaluate the integrity of the GJA intraoperatively including methylene blue testing, pneumatic insufflation, and endoscopic evaluation. The methylene blue leak test has been well described and used in bariatric procedures since 1980s [15]. A more recent study that utilized endoscopy to detect intraoperative leaks in 182 patients that underwent LRYGB detected an intraoperative leak rate of 10 %, although the methylene blue test was consistently negative in 61 patients [16]. Furthermore, endoscopy proved efficient in detecting correctable technical errors allowing surgeons to reinforce and retest the leak site intraoperatively, thus reducing postoperative morbidity with one study reporting 0 % leak rate in 290 patients using intraoperative EGD [3, 5, 17].

In our series, 80 (3.5 %) patients had air leak during endoscopy. Only 46 (2.0 %) of those occurred at low insufflation pressure and required suture line reinforcement. Postoperative leaks occurred in four (0.2 %) patients, of whom two had initial intraoperative leaks. Intraoperative endoscopy does carry small false-negative predictive value since it did not suggest leaks in two of our patients. Some diligence and persistence with slight manipulation of the anastomotic structures to avoid falsely negative intraoperative endoscopy is required. Nevertheless, it can be speculated that intraoperative endoscopic evaluation of GJA allowed the reduction of potential leak rate by 91.8 % compared to the no testing (no EGD) approach. The remaining 34 patients (1.5 %) had transient, non-reproducible air leak and none of them developed a clinical leak. Similar non-reproducible leaks were previously reported by Kligman in 12 patients and none of these patients developed a postoperative leak [18]. Furthermore, the surgeon should be aware that one or two bubbles might appear when the jejunal limb at the gastrojejunal anastomosis is insufflated due to displacement of air behind the limb leading to a transient leak. In two cases, intraoperative endoscopy identified correctable technical mistakes with GJA obstruction, thus further reducing postoperative morbidity.

GJA stricture rates in LRYGB have been reported to be between 4.9 and 11.1 % [6, 17, 19]. With the circular stapler technique, the stricture rate has been reported as high as 9.4–23 % [20, 21].On the other hand, the linear stapler technique was associated with lower stricture rate and morbidity [22]. Furthermore, a recent meta-analysis of 1,321 patients who underwent LRYGB revealed a significantly decreased GJA stricture rate with the linear stapler vs the circular stapler technique (RR 0.38; 95 % CI, 0.22–0.67; *P* = 0.0008) as well as decreased operative time and wound infection [23]. In this study, there were only early strictures at a rate of 1.1 % which is considerably lower than previously reported by other researchers and there were no delayed strictures at 1 year of follow-up.

The routine use of the endoscope will add an average of 5–10 min to the procedure time with low associated morbidity. Three (0.1 %) patients in this series sustained iatrogenic injury at the time of intraoperative endoscopy. In an additional three patients, the endoscopy was not completed due to failure to intubate the esophagus. Hence, the failure rate of intraoperative endoscopy to reduce the rate of GJA morbidity is in the range of 0.26 % while the estimated morbidity could have been 3.2 % in all study patients. Thus, intraoperative endoscopy is reasonably safe and reduces GJA-related morbidity from the expected 3.2 % to an actual frequency of 1.3 %.

While we agree with ASMBS statement that the majority of leaks will occur in patients who had a technically sound procedure [2], in our opinion, intraoperative endoscopic evaluation is helpful in minimizing the rate of leaks and strictures by identifying technical errors intraoperatively when they can be easily corrected without additional postoperative morbidity.

## Conclusion

The routine use of intraoperative endoscopy in LRYGB with the linear stapler anastomosis technique is associated with morbidity/failure rate of 0.26 % and low rate of GJA-related morbidity (leak rate of 0.2 % and stricture rate of 1.1 %). It is believed that endoscopic intraoperative evaluation of the GJA reduces the potential rate of anastomotic complications by more than 50 %.
